# Supplementary material for: Predicting Return to Work Following Myocardial Infarction: A Prospective Longitudinal Cohort Study
Source: Int J Environ Res Public Health. 2022 Jun 30;19(13):8032. doi: 10.3390/ijerph19138032 (PMC9266191; doi:10.3390/ijerph19138032)
Supplement: Supplementary file 1 [file ijerph-19-08032-s001.zip › ijerph-1775885-supplementary.pdf]

Supplementary Table S1. Results of the binary logistic analysis.

		B coefficient	Standard error	Wald test	Degrees of freedom	*p* value	Exponentiation of the B coefficient	Odds ratio	Confidence interval (95%) for odds ratio	
									Lower	Upper
Step 1	Smoking	1.506	.671	5.034	1	.025	4.508	4.508	1.210	16.796
	Age	-.053	.026	4.235	1	.040	.949	.949	.902	.997
	Gender	1.901	.785	5.862	1	.015	6.696	6.696	1.437	31.210
	Educational level			4.376	3	.224				
	Education group 1	-1.829	.985	3.450	1	.063	.161	.161	.023	1.106
	Education group 2	.207	.810	.066	1	.798	1.230	1.230	.252	6.014
	Education group 3	-.246	.606	.165	1	.684	.782	.782	.238	2.564
	Social support	.005	.057	.007	1	.935	1.005	1.005	.899	1.123
	Hypertension	.406	.524	.601	1	.438	1.501	1.501	.537	4.195
	Diabetes	-.626	.604	1.074	1	.300	.535	.535	.164	1.747
	LVEF	.012	.024	.264	1	.607	1.012	1.012	.966	1.061
	Anxiety	.354	.168	4.458	1	.035	1.425	1.425	1.026	1.981
	Depression	-.511	.155	10.817	1	.001	.600	.600	.443	.813
	Constant	-2.517	3.091	.663	1	.416	.081	.081		

Abbreviations: LVEF: left ventricular ejection fraction.
